# Novel Crown Cement Containing Antibacterial Monomer and Calcium Phosphate Nanoparticles

**DOI:** 10.3390/nano10102001

**Published:** 2020-10-11

**Authors:** Rashed AlSahafi, Abdulrahman A. Balhaddad, Heba Mitwalli, Maria Salem Ibrahim, Mary Anne S. Melo, Thomas W. Oates, Hockin H.K. Xu, Michael D. Weir

**Affiliations:** 1 Program in Dental Biomedical Sciences, School of Dentistry, University of Maryland, Baltimore, MD 21201, USA; rashed.alsahafi@umaryland.edu (R.A.); aabalhaddad@umaryland.edu (A.A.B.); hmitwalli@umaryland.edu (H.M.); 2 Department of Restorative Dental Sciences, College of Dentistry, Umm Al-Qura University, Makkah 24211, Saudi Arabia; 3 Department of Restorative Dental Sciences, College of Dentistry, Imam Abdulrahman Bin Faisal University, Dammam 31441, Saudi Arabia; 4 Department of Restorative Dental Sciences, College of Dentistry, King Saud University, Riyadh 11451, Saudi Arabia; 5 Department of Preventive Dental Sciences, College of Dentistry, Imam Abdulrahman Bin Faisal University, Dammam 31441, Saudi Arabia; m.s.y.ibrahim@gmail.com; 6 Department of General Dentistry, Division of Operative Dentistry, School of Dentistry, University of Maryland, Baltimore, MD 21201, USA; mmelo@umaryland.edu; 7 Department of Advanced Oral Sciences and Therapeutics, Biomaterials & Tissue Engineering Division, School of Dentistry, University of Maryland, Baltimore, MD 21201, USA; toates@umaryland.edu; 8 Center for Stem Cell Biology & Regenerative Medicine, School of Medicine, University of Maryland, Baltimore, MD 21201, USA; 9 Marlene and Stewart Greenebaum Cancer Center, School of Medicine, University of Maryland, Baltimore, MD 21201, USA

**Keywords:** calcium phosphate nanoparticles, antibacterial monomer, dental caries, crown cement, oral biofilms, *Streptococcus mutans*

## Abstract

Oral biofilm accumulation at the tooth–restoration interface often leads to recurrent dental caries and restoration failure. The objectives of this study were to: (1) develop a novel bioactive crown cement containing dimethylaminohexadecyl methacrylate (DMAHDM) and nano-sized amorphous calcium phosphate (NACP), and (2) investigate the mechanical properties, anti-biofilm activity, and calcium (Ca^2+^) and phosphate (PO_4_^3−^) ion release of the crown cement for the first time. The cement matrix consisted of pyromellitic glycerol dimethacrylate and ethoxylated bisphenol-A dimethacrylate monomers and was denoted PEHB resin matrix. The following cements were tested: (1) RelyX luting cement (commercial control); (2) 55% PEHB + 45% glass fillers (experimental control); (3) 55% PEHB + 20% glass + 25% NACP + 0% DMAHDM; (4) 52% PEHB + 20% glass + 25% NACP + 3% DMAHDM; (5) 51% PEHB + 20% glass + 25% NACP + 4% DMAHDM; (6) 50% PEHB + 20% glass + 25% NACP + 5% DMAHDM. Mechanical properties and ion release were measured. *Streptococcus* *mutans* (*S. mutans*) biofilms were grown on cements, and colony-forming units (CFUs) and other biofilm properties were measured. The novel bioactive cement demonstrated strong antibacterial properties and high levels of Ca^2+^ and PO_4_^3−^ ion release to remineralize tooth lesions. Adding NACP and DMAHDM into the cement did not adversely affect the mechanical properties and dentin bonding strength. In conclusion, the novel NACP + DMAHDM crown cement has excellent potential for restoration cementation to inhibit caries by suppressing oral biofilm growth and increasing remineralization via Ca^2+^ and PO_4_^3−^ ions. The NACP + DMAHDM composition may have wide applicability to other biomaterials to promote hard-tissue formation and combat bacterial infection.

## 1. Introduction

Secondary caries at the margins of fixed dental restorations have been reported as the most common cause of failure in such treatment [1]. In most clinical events, secondary caries at crown margins leads to interventional approaches involving crown removal and replacement, which affect the cost-effectiveness and cost benefit of oral healthcare, with an impact on both the individual patient as well as on the community caries level [2]. The tooth-restoration interface is a critical area for secondary caries. The tooth-restoration interface is a complex environment with a dynamic equilibrium in substances and minerals present in saliva and hard tooth tissues. When the equilibrium shifts to an acidic environment, the tooth structure will start to demineralize in a way similar to primary caries [3]. Characteristics that increase the accumulation of food debris or limit its cleaning can be considered as potential risk factors for secondary caries, which is likely why secondary caries mainly occur at the tooth-restoration interface [4].

Secondary caries at the margins of dental restorations are one of the most critical aspects affecting the survivability of the restorations. Creating an ideal marginal adaptation of the crown restoration is currently a significant challenge [5]. Restoration marginal discrepancy may expose dental cement to the oral environment, thus increasing the risk of demineralization attack [6]. Additionally, the marginal gap increases the potential for food accumulation around the crown restoration and limits the cleaning ability. This challenge makes it easier for the bacteria to penetrate the tooth-cement interface and for plaque to build up around the restoration margin, which changes the equilibrium of the oral environment and leads to the initiation of secondary caries. However, even when the marginal gap is clinically acceptable, other risk factors such as the subgingival location of the margins or other host-related factors may increase the risk of secondary caries. Indeed, secondary caries were the most common reason for restoration failure and replacement [3,7,8]. For example, a study found that 38% of all crown failures and replacements were due to secondary caries [9]. While there is no clear understanding of whether different restoration types have different infected dentin forms [10,11], it appears that infected dentin that originates from primary and secondary caries have comparable microbial profiles, including mainly anaerobic *Streptococcus mutans* (*S. mutans*) and *Lactobacilli* [12].

Saliva has numerous immune factors that demonstrate an important function against oral microorganisms. Saliva quantity and quality could also modify an individual’s risk for secondary caries by inducing buffering and washing capacities. Salivary fluid can neutralize the pH and mechanically clean the teeth in the oral environment. Patients diagnosed with xerostomia are associated with a higher risk for secondary caries and all forms of dental caries [13,14]. However, secondary caries may develop in the presence of a cariogenic biofilm, despite patient-related factors and the various qualities and types of dental restorations [3]. Consequently, designing a crown cement containing an antibacterial monomer and calcium phosphate nanoparticles to reduce caries and to regenerate tooth minerals would be useful to overcome this clinical issue.

Dental cement acts as the connection between the tooth structure and the crown material. The ideal dental cement should be biocompatible and antibacterial, and have a low film thickness, low viscosity, minimal solubility, sufficient working time, and no microleakage. In addition, it should have comparable strength and stiffness to dentin [5,6].

From a clinical point of view, nanoparticles of amorphous calcium phosphate (NACP) may present the advantage of reducing the initial formation of secondary caries by providing long-lasting release of calcium (Ca^2+^) and phosphate (PO_4_^3−^) ions. NACP enhances the precipitation and deposition of minerals into tooth structures [15]. Additionally, bacterial acids and the local pH can be neutralized by NACP [16,17,18]. NACP is a non-crystalline structure with a relatively high surface area of 17.76 m^2^/g compared to the conventional CaP with a 0.5 m^2^/g surface area [15]. Resin-based materials with NACP release Ca^2+^ and PO_4_^3−^ ions similar to conventional CaP resin-based materials with improved mechanical properties [17,19]. The addition of NACP is a practical method to provide the oral environment with a continuous release of calcium and phosphate ions triggered only by pH drop. In human in situ analysis, resin-based materials with NACP showed four times the mineral regeneration in the tooth structure compared to commercial fluoride-releasing resin-based materials [16]. In another investigation, tooth demineralization adjacent to resin-based materials without NACP was three times higher compared to resin-based materials containing NACP [20].

Another approach to reduce biofilm formation at the tooth-restoration interface is by the use of contact-killing monomers, such as quaternary ammonium methacrylates (QAMs), within the resin matrix of resin-based materials [21]. The positively charged quaternary amine N^+^ of QAMs can disrupt the bacterial membrane, change the essential ion balance (i.e., Na^+^, K^+^, Mg^2+^, and Ca^2+^) and cause cytoplasmic leakage by direct binding to the negatively charged bacterial cell membrane [22]. For short-chained QAMs, the antimicrobial activity relies only on the positively charged ammonium group. On the other hand, long-chained QAMs have double-killing properties: (1) positively charged quaternary amine N^+^; and (2) increased hydrophobicity due to the increased alkyl chain length of QAMs, which may enhance their ability to penetrate the hydrophobic bacterial cell membrane [23,24]. Dimethylaminohexadecyl methacrylate (DMAHDM), with an alkyl chain length of 16, has been shown to have the most effective antibacterial properties. A further increase to the chain length was shown to decrease the antibacterial efficiency of the positively charged quaternary ammonium groups as too long chain lengths may bend to form barriers between bacteria and positively charged quaternary amine N^+^ [24].

In recent studies, DMAHDM was incorporated into resin-based materials and exhibited an excellent antimicrobial activity [25,26,27]. It has been reported that DMAHDM is a positively charged monomer with the ability to work through direct contact killing mechanisms by interacting with negatively charged bacterial cell membranes, causing bacterial cell disturbance and lysis [28,29]. DMAHDM and NACP were incorporated into dental adhesives, pit and fissure sealants, and resin composites in previous studies. They presented strong antibacterial outcomes with a high level of Ca^2+^ and PO_4_^3−^ ion release [20,25,30,31]. Consequently, the incorporation of DMAHDM and NACP into a resin-based material could help to prevent dental caries through DMAHDM contact inhibition action and tooth remineralization through the release of Ca^2+^ and PO_4_^3−^ ions into the tooth-restoration interface [25,32]. However, up to the present time, there have been no reports on the incorporation of DMAHDM and NACP into a resin-based crown cement to achieve strong, long-lasting antibacterial and Ca^2+^ and PO_4_^3−^ ion release abilities.

The aim of this study was to produce a novel resin-based crown cement containing DMAHDM and NACP. We hypothesize that the contact-killing mechanism provided by DMAHDM and the remineralization capabilities of the NACP fillers would provide a potential clinical approach to control the onset of secondary caries at the tooth-restoration interface without adversely affecting the mechanical properties of the resin-based cement.

## 2. Materials and Methods

### 2.1. Experimental Design

Novel dual cure crown cement was formulated using pyromellitic glycerol dimethacrylate (PMGDM, Esstech, Essington, PA, USA), ethoxylated bisphenol-A-dimethacrylate (EBPADMA, Sigma-Aldrich, St. Louis, MO, USA), hydroxyethyl methacrylate (HEMA, Esstech, Essington, PA, USA), and bisphenol A-glycidyl methacrylate (BisGMA) (Esstech). Camphorquinone (CQ, Sigma-Aldrich) and ethyl 4-*N*,*N*-dimethylaminobenzoate (4E, Sigma-Aldrich) were added as photoinitiators. Cumene hydroperoxide (CHP, Sigma-Aldrich) and benzoylthiourea (BTU, Sigma-Aldrich) were added as chemical initiator and accelerator, and 2,6-ditertbutyl-4-methylphenol (BHT, Sigma-Aldrich) as a stabilizer. The formulated mix of monomers was referred to as PEHB. Each experiment group had two pastes: paste A and paste B at a 1:1 mass ratio, for detailed information, see Table 1, Table 2, Table 3 and Table 4. PEHB at a filler:matrix mass ratio of 45:55 was used. Then, the following resin cements were tested:

(1) **RelyX luting cement** (3M, St Paul, MN, USA) (referred to as “Commercial control”) (Table 1);
(2) **55% PEHB + 45% glass fillers** (referred to as “Experimental control”) (Table 2);
(3) **55% PEHB + 20% glass fillers + 25% NACP** + **0% DMAHDM** (referred to as “Glass + NACP + 0% DMAHDM”) (Table 2);
(4) **52% PEHB + 20% glass fillers + 25% NACP + 3% DMAHDM** (referred to as “Glass + NACP + 3% DMAHDM”) (Table 3);
(5) **51% PEHB + 20% glass fillers + 25% NACP + 4% DMAHDM** (referred to as “Glass + NACP + 4% DMAHDM”) (Table 4);
(6) **50% PEHB + 20% glass fillers + 25% NACP + 5% DMAHDM** (referred to as “Glass + NACP + 5% DMAHDM”) (Table 5).

Different percentages of BisGMA were tested in the shear bond strength test as follows:

- Formula A contains 4.85% BisGMA;
- Formula B contains 5% BisGMA;
- Formula C contains 6% BisGMA.

### 2.2. Synthesis of DMAHDM Monomer and NACP Fillers

A spray-drying technique was used to synthesize NACP. Calcium carbonate and dicalcium phosphate anhydrous were dissolved in acetic acid to create calcium (Ca^2+^) and phosphate (PO_4_^3−^) with concentrations of 8 mmol/L and 5.333 mmol/L, correspondingly. The final molar ratio of Ca^2+^/PO_4_^3−^ of 1.5 was sprayed into a high temperature chamber of the spray-drying machine to vaporize the water and volatile acid. Dry NACP was collected using an electrostatic precipitator. NACP with a mean particle size of approximately 142 nm was the final product [19].

A modified Menschutkin reaction was used to produce DMAHDM, as explained previously [33]. Briefly, 10 mmol of 1-bromohexadecane (TCI America, Portland, OR, USA), 10 mmol of 2-(dimethylamino)ethyl methacrylate (Sigma-Aldrich), and 3 g of ethanol were mixed in a 20 mL scintillation vial. Stirring was done at 70 °C for 24 h. Then, DMAHDM was collected after evaporating the solvent [34].

### 2.3. Characterization of NACP

Transmission electron microscopy (TEM, Tecnai T12, FEI, Hillsboro, OR, USA) was used to evaluate the nanoparticles’ size. Nanoparticles were placed on a perforated copper grid coated by a carbon film. The sample was ultrasonicated for 30 min in acetone before deposition, to avoid particle agglomeration. Images were acquired using AMT V600 software and particle sizes of individual particles were measured with the same software.

### 2.4. Dentin Shear Bond. Strength Testing

Extracted human molars (*n* = 10 for each group) were stored in 0.01% thymol solution at 4 °C. Teeth were mounted vertically in an Ultradent mold (Ultradent Products, South Jordan, UT, USA) using self-cure polyethyl methacrylate diethyl phthalate (PMDP) acrylic resin (Esschem, Linwood, PA, USA). Teeth occlusal surfaces were removed by a model trimmer until mid-coronal dentin was revealed. Then, teeth were polished to a final coarseness of 120-, 320-, or 600-grit with silicon carbide (SiC) abrasive paper. The dentin surface was etched with 37% phosphoric acid (ScotchBond Etchant, 3M, St. Paul, MN, USA) for 15 s, then rinsed with water for 15 s. The primer consisted of PMGDM and HEMA at a mass ratio 3.3/1, with 50% acetone solvent. The primer was applied with a brush-tip applicator. After 20 s, an adhesive was applied which consisted of 44.5% PMGDM, 39.5% EBPADMA, 10% HEMA, and 5% BisGMA with 0.8% 4E and 0.2% CQ as a photoinitiator [35], then an Ultradent insert was used to build resin cement cylinders of 2.37 mm in diameter bonded on the test surface. After 80 s from starting the cement mixing, the resin cement cylinders were light cured (Labolight, DUO, GC, Tokyo, Japan) for 60 s with a radiance emittance of 1200 mW/cm^2^. Samples were stored in distilled water for 24 h. A universal testing machine (MTS Insight 1, Cary, NC, USA) was used with a load applied at a crosshead speed of 0.5 mm/min until bond failure. Dentin shear bond strength was calculated using the following equation: 4 P/(πd^2^), where P is the load at failure and d is the diameter of the composite [30].

### 2.5. Flexural Strength and Elastic Modulus

Resin cement samples for flexural strength and elastic modulus tests were made using 2 × 2 × 25 mm stainless steel molds. Cement paste A and paste B were mixed equally then placed into the mold which was covered with Mylar strips and glass slides on both open sides of the mold. After 80 s from starting cement mixing, a light-curing unit (Labolight) with a radiance emittance of 1200 mW/cm^2^ was used for two minutes at each side. The samples were stored for 24 h at 37 °C. Then, the samples were further stored in water for 24 h at 37 °C.

Flexural strength and elastic modulus were calculated using three-point flexure with a 10 mm span at a crosshead speed of 1 mm/min on a computer-controlled universal testing machine (MTS Insight 1) by the following equations:

(1) $$S=\frac{3PL}{2bh^{2}}$$

S = flexural strength, P_max_ = fracture load, L = the span, b = specimen width, and h = specimen thickness.

(2) $$E=\frac{P}{d}\times \frac{L^{3}}{4bh^{3}}$$

E = elastic modulus.

The load P divided by displacement d is the slope in the linear elastic region [32].

### 2.6. Film Thickness

Two optically flat square glass plates with a contact surface area of 200 ± 10 mm^2^ were used. Each glass plate has a uniform thickness of 5 mm. The combined thickness of the two glass plates was measured (reading A). A portion of the resin cement was placed onto the center of the lower plate, and the other glass plate was applied on the cement. A load of 150 N was applied vertically and centrally to the cement sample via the top plate for (180 ± 10) s [36]. Then, the thickness of the two glass plates and the interposed film of cement was measured using a micrometer (reading B). The difference between reading A and reading B was recorded to the nearest micrometer as the thickness of the resin cement [37].

### 2.7. Measurement of Initial Calcium and Phosphate Ions Release

To simulate the cariogenic condition in the oral cavity, 133 mmol/ L of a sodium chloride (NaCl) solution was buffered to pH 4 with 50 mmol/L lactic acid [19,38]. For each experimental group, three specimens of approximately 2 × 2 × 12 mm were immersed in 50 mL of solution to generate a specimen volume/solution of approximately 2.9 mm^3^/mL. Six tubes for each group were kept in a 37 °C incubator during the experiment. The Ca^2+^ and PO_4_^3−^ ion concentrations released from the specimens were measured at 1, 3, 5, 7, 14, 21, 28, 35, and 42 days. At each specific time, 0.5 mL of the solution was taken from each tube and replaced with fresh solution. The incubating solution pH was maintained and adjusted to pH 4 throughout the experiment time. The collected solutions were analyzed for Ca^2+^ and PO_4_^3−^ concentrations using known standards and calibration curves via a spectrophotometric method (SpectraMax M5, Molecular Devices LLC, San Jose, CA, USA) [31].

### 2.8. S. mutans Biofilm Model

#### 2.8.1. Sample Preparation

Circular molds with a diameter of 8 mm and a thickness of 1 mm were used to make cement disks. Each sample was light cured (1200 mW/cm^2^; 60 s; Labolight DUO, GC America, Alsip, IL, USA) on each surface and incubated in 37 °C incubator for 24 h. Then samples were immersed and stirred at 100 rpm in distilled water for 1 h to remove unreacted monomers [22]. Then, specimens were sterilized using ethylene oxide (Anprolene AN74i, Andersen, Haw River, NC, USA). To ensure the complete release of entrapped ethylene oxide, samples were de-gassed for seven days [23].

#### 2.8.2. *S. mutans* Biofilm Formation

*S. mutans* UA159 (ATCC, Manassas, VA, USA) was cultured overnight (16–18 h) in brain-heart infusion broth (BHI, Sigma-Aldrich) at 37 °C and 5% CO_2_. The resulting bacterial suspension was adjusted to an optical density of 0.9 at 600 nm and diluted 20-fold in biofilm medium containing BHI broth supplemented with 2% sucrose (by mass) to prepare the inoculum. Samples were placed in the wells of 24-well plates and immersed in 1.5 mL of the inoculum. The samples were incubated at 37 °C in 5% CO_2_, and fresh medium was added after 24 h of the incubation. At a total of 48 h incubation, the samples were moved to a perform colony-forming unit (CFU) test [25,39].

#### 2.8.3. Colony-Forming Unit Counts

After the cement samples (*n* = 4) had been incubated for 48 h, each sample with the attached biofilm was washed in 1 mL of phosphate-buffered saline (PBS), moved to a vial containing 1 mL of PBS, sonicated for 7 min, and vortexed for 5 s at maximum speed to collect the biofilm. The bacterial suspensions were serially diluted (10^1^–10^6^-fold) and transferred into each BHI agar plate to calculate the total number of colonies. The agar plates were incubated at 37 °C in 5% CO_2_ for 48 h [18]. The results were calculated based on the number of CFUs and the dilution factor; log10 transformed data are expressed as CFU/sample.

#### 2.8.4. Live/Dead Staining of Biofilms

Cement disks with 48 h biofilms were washed with PBS to remove planktonic bacteria. Following the manufacturer’s instructions, cement disks were stained with the BacLight live/dead kit (Molecular Probes, Eugene, OR, USA). An equal mixture of 2.5 μM SYTO 9 and 2.5 μM propidium iodide was used to stain each sample for 15 min. Green fluorescence showed the presence of live bacteria stained with SYTO9. Meanwhile, bacteria with defective membranes were stained with propidium iodide to emit a red fluorescence. An inverted epifluorescence microscope (Eclipse TE2000-S, Nikon, Melville, NY, USA) was used to examine the stained biofilm disks [40].

#### 2.8.5. MTT Assay for Quantification of Metabolic Activity of Biofilms

Metabolic activity of biofilms was measured by a 3-[4,5-dimethylthiazol-2-yl]-2,5-diphenyltetrazolium bromide (MTT) assay. MTT is a colorimetric assay that measures the enzymatic reduction of a yellow tetrazole to formazan. Cement disks with 2-day biofilms were transferred to a new 24-well plate filled with 1 mL of MTT dye (0.5 mg/mL MTT in PBS) and then incubated at 37 °C in 5% CO_2_. After 1 h, the disks were moved to a new 24-well plate with 1 mL of dimethyl sulfoxide (DMSO) to solubilize the formazan crystals, and the plate was incubated in the dark for 20 min at room temperature. After mixing by pipetting, 200 μL of the DMSO solution were obtained from each specimen and transferred to a 96-well plate, and the absorbance at 540 nm was measured by a microplate reader (SpectraMax M5). A higher absorbance is correlated to a higher formazan concentration, which indicates a greater metabolic activity in the biofilm on the disk [41].

### 2.9. Statistical Analysis

A Shapiro-Wilk test was performed to confirm the normality and equal variance of data. One-way analyses of variance (ANOVA) and Tukey’s comparison tests were performed to detect the significant effects of the dependent variables on the mechanical properties and antibacterial effects. A *p*-value of <0.05 was considered statistically significant. All the statistical analyses were performed using Sigma Plot (SYSTAT, Chicago, IL, USA).

## 3. Results

### 3.1. Characterization of NACP

**Figure 1:**
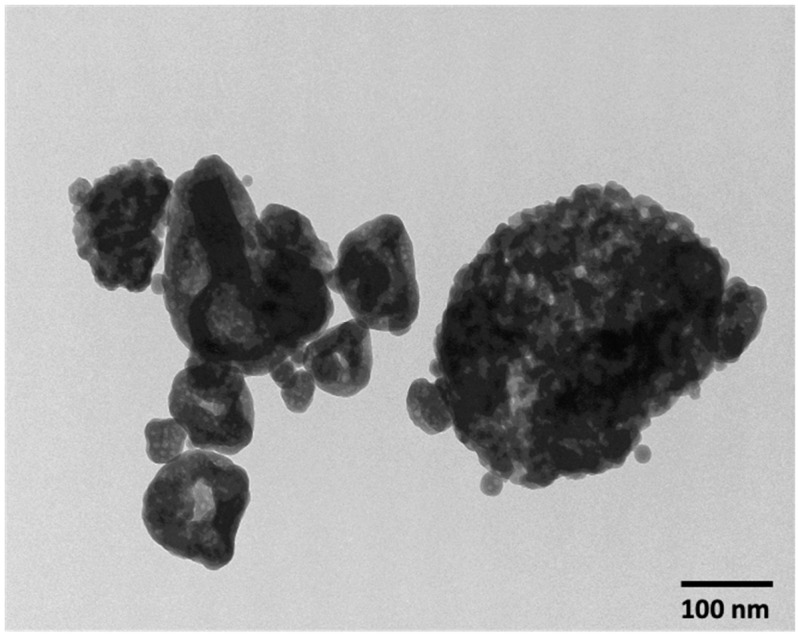
TEM of nano-sized amorphous calcium phosphate (NACP) synthesized via spray-drying technique and collected using an electrostatic precipitator.

A representative TEM image of nano-sized amorphous calcium phosphate (NACP) is shown in Figure 1. The nanoparticle size distribution ranged from 49 nm to 312 nm, with a mean particle size of 142 nm.

### 3.2. Dentin Shear Bond. Strength

**Figure 2:**
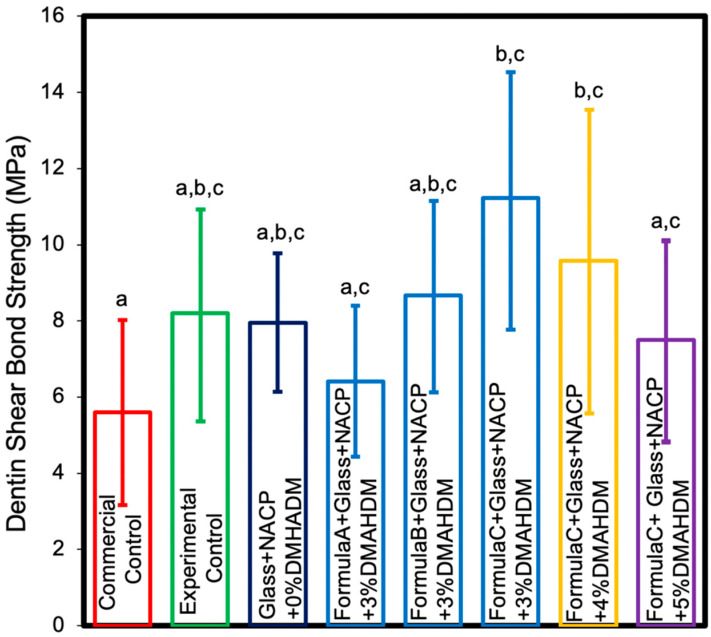
Dentin shear bond strengths (mean ± SD; *n* = 10). Adding 5% DMAHDM combined with 25% NACP demonstrated comparable shear bond strength to the commercial control (*p* > 0.05). Adding 3% and 4% DMAHDM to PEHB with 6% BisGMA combined with 25% NACP demonstrated significantly higher shear bond strength compared to the commercial control (*p* < 0.05). Values indicated by different letters are statistically different from each other (*p* < 0.05).

The dentin shear bond strength results are shown in Figure 2 (mean ± SD; *n* = 10). Adding 25% of NACP filler to the cement matrix showed no significant effect on dentin shear bond strength (*p* > 0.05) compared to the experimental control. Increasing the BisGMA percentage from 4.85% to 6% in the cement monomers demonstrated enhanced dentin shear bond strength (*p* < 0.05). Based on this outcome, the remaining experiments in this study were conducted with 6% BisGMA and the formulations containing 4.85% and 5% BisGMA were excluded. Adding 3% and 4% DMAHDM to cement monomers with 6% BisGMA combined with 25% NACP demonstrated significantly higher shear bond strength compared to the commercial control (*p* < 0.05). Adding 5% DMAHDM combined with 25% NACP demonstrated comparable shear bond strength to the commercial control (*p* > 0.05). These results indicate that increasing the DMAHDM concentration and adding the NACP did not significantly reduce dentin shear bond strengths of the resin crown cement. On the other hand, changing the BisGMA concentration from 4.85% to 6% led to a significant increase in dentin shear bond strengths of the resin crown cement.

### 3.3. Flexural Strength

**Figure 3:**
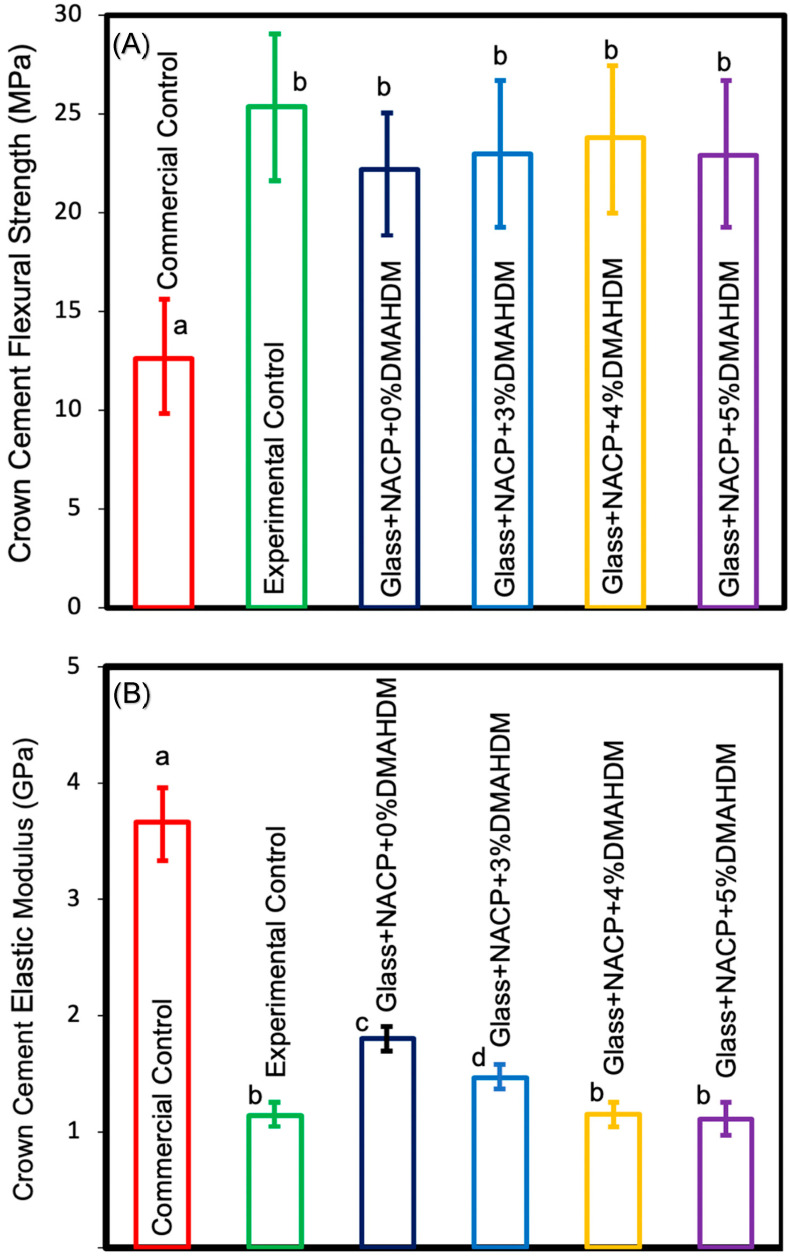
(**A**) Flexural strength and (**B**) elastic modulus (mean ± SD; *n* = 10). Adding 3%, 4%, and 5% DMAHDM combined with 25% NACP resulted in higher flexural strength values compared to the commercial control (*p* < 0.05). The incorporation of 4% and 5% DMAHDM combined with 25% NACP in the resin cement resulted in comparable elastic modulus values to the experimental control (*p* > 0.05). The incorporation of 25% NACP and 3% DMAHDM combined with 25% NACP in the resin cement resulted in higher elastic modulus values compared to the experimental control (*p* > 0.05). The elastic modulus values of the commercial control group were significantly greater than all other groups (*p* < 0.05). Values indicated by different letters are statistically different from each other (*p* < 0.05).

Flexural strength and elastic modulus results of the cement are shown in Figure 3 (mean ± standard deviation (SD); *n* = 10). The incorporation of 3%, 4%, and 5% DMAHDM combined with 25% NACP in the resin cement resulted in higher flexural strength values compared to the commercial control (*p* < 0.05). Experimental control resin cement revealed the highest flexural strength among the experiment groups with a statistically significant difference compared to the commercial control (*p* < 0.05). However, it showed comparable results to other groups (*p* > 0.05). These outcomes demonstrate that increasing the DMAHDM concentration and adding the NACP did not significantly reduce the flexural strength of the resin crown cement.

The elastic modulus values of the commercial control group were significantly greater than all other groups (*p* < 0.05). The incorporation of 4% and 5% DMAHDM combined with 25% NACP in the resin cement resulted in comparable elastic modulus values to the experimental control (*p* > 0.05). The incorporation of 25% NACP and 3% DMAHDM combined with 25% NACP in the resin cement resulted in higher elastic modulus values compared to the experimental control (*p* > 0.05).

### 3.4. Film Thickness

**Figure 4:**
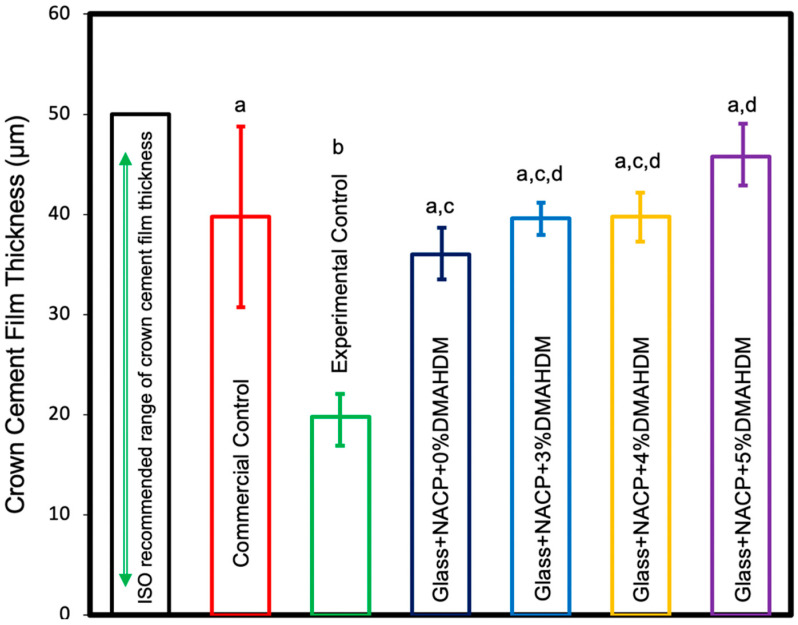
Cement film thickness (mean ± SD; *n* = 5). Adding 3%, 4%, and 5% DMAHDM combined with 25% NACP demonstrated film thickness within the International Organization for Standardization (ISO) and showed no significant difference compared to the commercial control (*p* > 0.05). Values indicated by different letters are statistically different from each other (*p* < 0.05).

The cement film thickness values are shown in Figure 4 (mean ± SD; *n* = 5). All cement groups had film thickness values that met the International Organization for Standardization (ISO) requirement. The experimental control resin cement revealed the lowest film thickness among the experiment groups with a statistically significant difference compared to the 5% DMAHDM group (*p* < 0.05). However, the 5% DMAHDM group showed comparable results to other groups (*p* > 0.05). Resin cements with 3% DMAHDM, 4% DMAHDM, and 5% DMAHDM demonstrated comparable film thickness values to the commercial control group (*p* < 0.05). These outcomes demonstrate that increasing the DMAHDM concentration and adding 25% NACP did not significantly increase the crown cement film thickness. All cement groups had film thickness values that met the ISO requirement.

### 3.5. Initial Calcium and Phosphate Ion. Release

**Figure 5:**
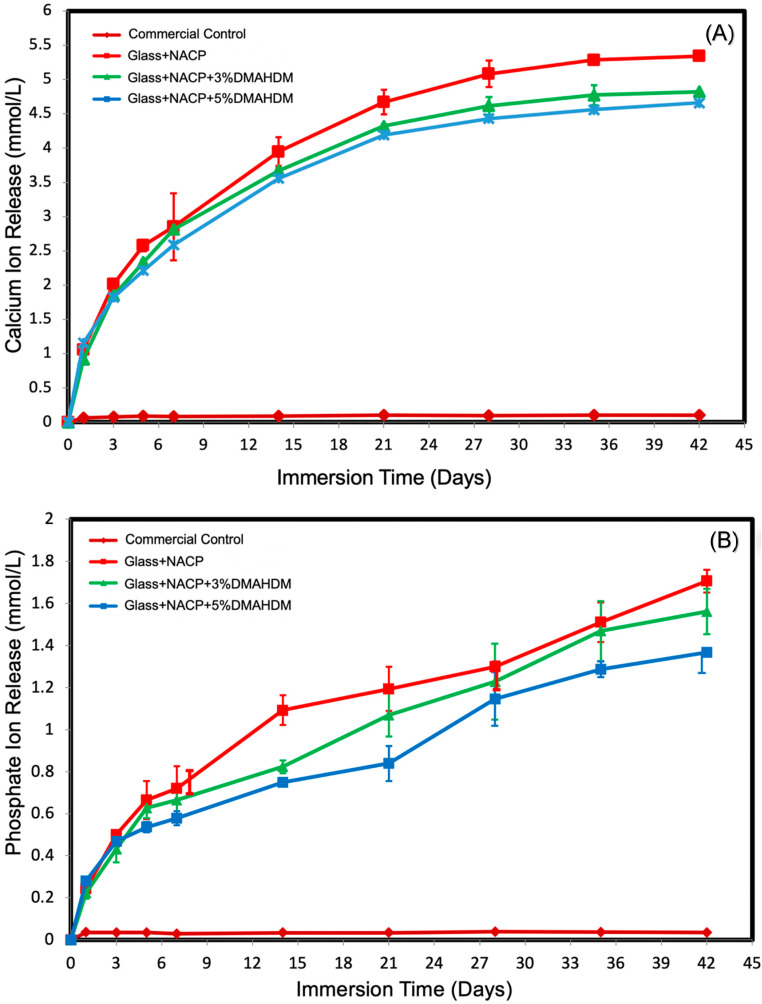
Initial ion release from the virgin cement samples. (**A**) Ca^2+^ ion release and (**B**) PO_4_^3−^ ion release (mean ± SD; *n* = 6). All tested experimental cement groups had high Ca^2+^ and PO_4_^3−^ ion releases over 42 days. Increasing the DMAHDM percentage from 3% to 5% did not significantly affect Ca^2+^ and PO_4_^3−^ ion release (*p* < 0.05).

The initial Ca^2+^ and PO_4_^3−^ ion release from cement specimens is plotted in Figure 5A,B (mean ± SD; *n* = 6). The ion concentrations for the experimental groups significantly increased with time from 1 to 42 days, indicating continuous ion release (*p* < 0.05). Increasing the DMAHDM level from 3% to 5% did not significantly affect Ca^2+^ and PO_4_^3−^ ion release (*p* < 0.05).

#### 3.5.1. Colony-Forming Unit Counts of *S. mutans* Biofilm

**Figure 6:**
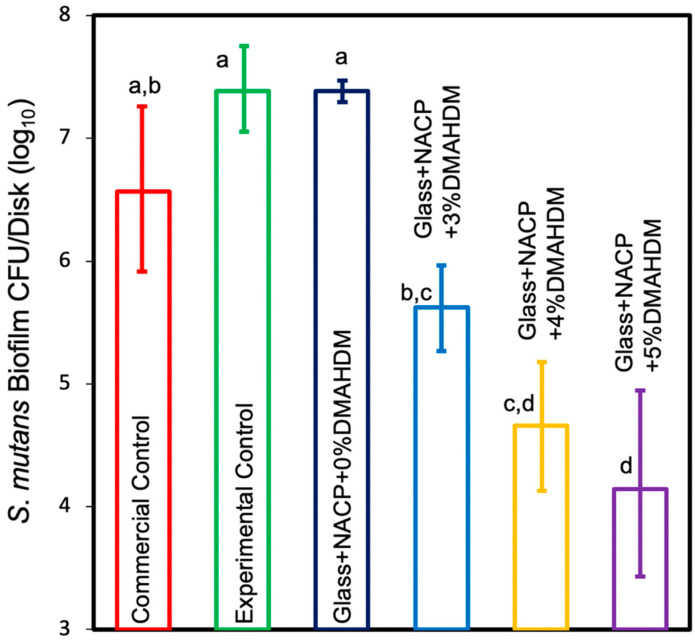
Colony-forming unit (CFU) counts for 2-day biofilms on resin cement disks (mean ± SD; *n* = 4). Adding 4% and 5% DMAHDM + NACP to resin cement significantly reduced CFU count for *Streptococcus mutans* compared to the commercial and experimental controls (*p* < 0.05). The incorporation of 3% DMAHDM + NACP in resin cement significantly reduced CFU count for *S. mutans* compared to the experimental control (*p* < 0.05). Values indicated by different letters are statistically different from each other *(p* < 0.05).

The CFU results of the *S. mutans* biofilm are shown in Figure 6. The incorporation of 4% and 5% DMAHDM + NACP in the resin cement formulations significantly reduced the CFU count by 2 and 2.5 log, respectively, compared to the commercial control (*p* < 0.05) and significantly reduced the CFU count by 3 and 3.5 log, respectively, compared to the experimental control (*p* < 0.05). The incorporation of 3% DMAHDM + NACP in resin cement significantly reduced the CFU count for *S. mutans* compared to the experimental control (*p* < 0.05). The incorporation of NACP alone was associated with a comparable *S. mutans* biofilm to the experimental control (*p* > 0.05). These results showed that increasing DMAHDM concentration to 5% demonstrated the highest biofilm reduction.

#### 3.5.2. MTT Assay of Metabolic Activity of *S. mutans* Biofilms

**Figure 7:**
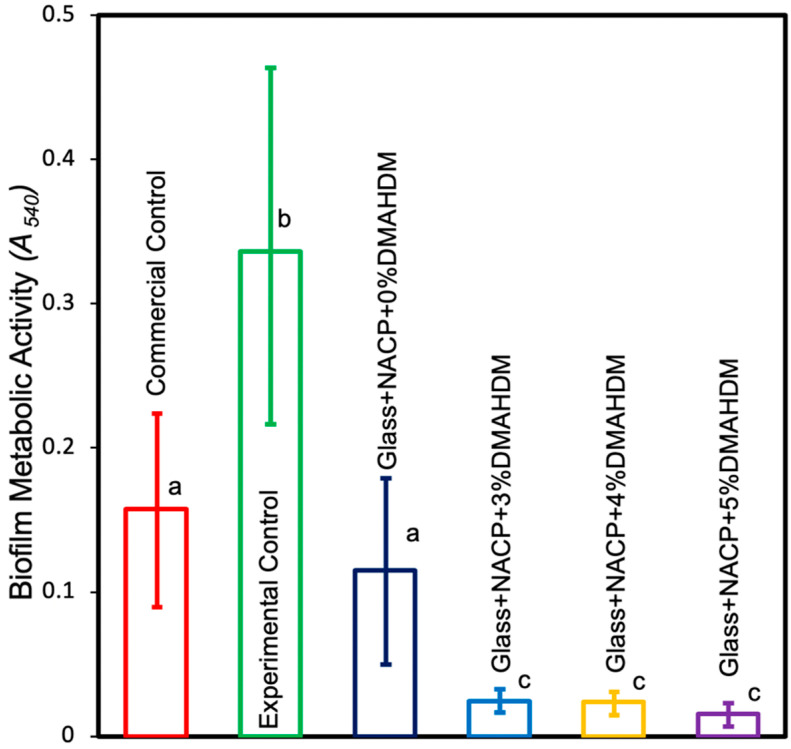
Metabolic activity for 2-day *S. mutans* biofilms on resin cement disks (mean ± SD; *n* = 4). The incorporation of different percentages of DMAHDM combined with NACP was more effective in reducing the metabolic activities than the incorporation of NACP alone (*p* < 0.05). Values indicated by different letters are statistically different from each other *(p* < 0.05).

The two-day biofilm bacterial metabolic activity on cement samples is shown in Figure 7. The experimental groups significantly reduced the metabolic activities compared to the experimental control (*p* < 0.05). Metabolic activity was decreased from 0.33 (OD540/cm) for the experimental control, to 0.024 for 3% DMAHDM + NACP, 0.023 for 4% DMAHDM + NACP, and 0.015 for 5% DMAHDM + NACP (*p* < 0.05). The incorporation of DMAHDM with NACP was more effective in reducing the metabolic activities than the incorporation of NACP alone (*p* < 0.05).

#### 3.5.3. Live/Dead Staining of *S. mutans* Biofilms

**Figure 8:**
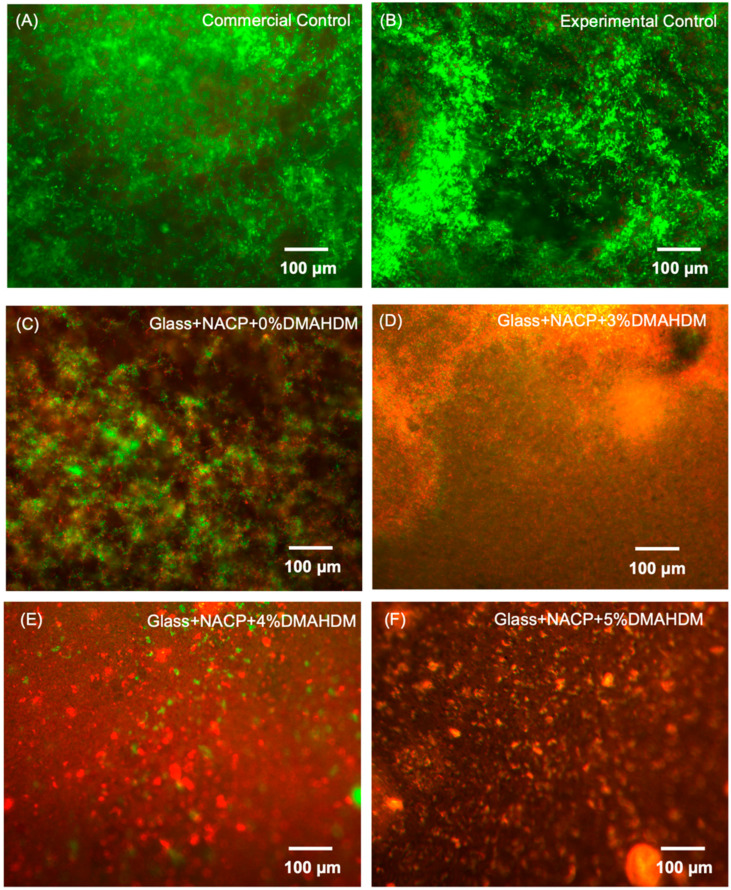
Demonstrative live/dead staining images of 2-day *S. mutans* biofilms: live bacteria were stained green, and compromised bacteria were stained red. Mixed live and dead bacteria produced yellow/orange colors. (**A**–**C**) Commercial, experimental control, and NACP+0%DMAHDM cements were mostly covered by live bacteria. (**D**–**F**) In contrast, NACP + DMAHDM cement had significant amounts of dead bacteria.

Live/dead images of 2-day biofilms are shown in Figure 8. Live bacteria are indicated with green stain, while compromised bacteria with disrupted membranes are shown in red. The experimental control resin cement was associated with more active bacteria. Fewer active bacteria were observed with increasing DMAHDM percentage combined with NACP fillers.

## 4. Discussion

In this study, a novel crown cement with dual antibacterial contact-killing and remineralization capabilities was designed. A literature search has shown no reports of such an approach to address the onset of caries at the tooth-restoration interface. The capacity of the cement to inhibit *S. mutans* biofilm while maintaining high levels of ion release was accomplished and the study hypotheses were confirmed. The incorporation of DMAHDM and NACP into the crown cement significantly reduced *S. mutans* biofilm growth while maintaining excellent mechanical properties. The new crown cement reduced *S. mutans* biofilm growth by two to three orders of magnitude. Furthermore, the bioactive cement has the potential to remineralize and enhance tooth structure through the high level of Ca^2+^ and PO_4_^3−^ release.

Antibacterial properties of contemporary dental cement have been studied broadly and it has been suggested that the low initial pH or the fluoride release of the cement could affect the bactericidal outcome. Nevertheless, there is no clear understanding of whether the low pH or low level of fluoride release is sufficient for a long-lasting antibacterial effect, especially as the onset of caries around the crown margins usually requires several days to years to develop [42,43]. The low initial pH of dental cement could have an undesirable outcome, as acid penetration through dentinal tubules can initiate pulpal irritation [44]. Additionally, the long-term success of fluoride-releasing dental cements is debatable due to their short-lasting effect and limited ability to re-release/recharge [45]. In recent reports to overcome these limitations, DMAHDM was combined into the resin matrix to maximize the antibacterial effect against cariogenic biofilms. DMAHDM has the ability to copolymerize with the resin matrix [40]. Accordingly, the antibacterial agent in the resin matrix is effectively immobilized by covalent bonds and is not lost or released over time, thereby providing long-lasting antibacterial effect.

**Figure 9:**
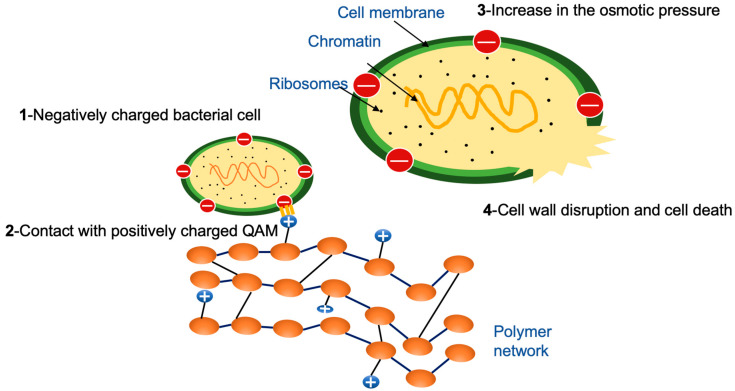
The antibacterial mechanism of DMAHDM. When the positively charged sites (N^+^) of DMAHDM contacts the negatively charged bacterial cell membrane, the electric balance of the cell membrane could be disturbed, leading to cytoplasmic leakage and bacterial cell death.

In previous studies, the antibacterial mechanism of DMAHDM was reported to work by contact inhibition (Figure 9). When the positively charged sites (N^+^) of DMAHDM contact the negatively charged bacterial cell membrane, the electric balance of the cell membrane could be disturbed, leading to cytoplasmic leakage and bacterial cell death [46]. The upper limit of 5% of DMAHDM in the cement was selected, following a prior report that presented strong antibacterial activity using 5% DMAHDM, without adversely affecting the physical and mechanical properties of the resin [47]. The biofilm CFU was reduced significantly when incorporating 3%, 4%, and 5% DMAHDM in the resin-based crown cement. It showed about a 2–3 log reduction in the activity of *S. mutans* biofilms [48]. This result is consistent with previous studies for oral biofilms exposed to resin-based pit and fissure sealants and resin bonding agents containing DMAHDM. In the present study, biofilm metabolic activities were significantly reduced when adding NACP with DMAHDM, compared to the experimental control. Moreover, adding NACP without DMAHDM showed a significant reduction in metabolic activity, compared to the experimental control. This could be attributed to the observation that the MTT assay results correlated with the physiological state of the viable bacterial cells. We observed a large number of viable microorganisms with a low rate of metabolic activity; in addition, we also observed a small number of viable microorganisms with a high rate of metabolic activity.

On the other hand, CFU assays correlated to the number of bacterial cell growth on the BHI agar plates, regardless of their metabolic activity rate. For example, a small number of microorganisms with a high rate of metabolic activity could give a lower CFU count, compared to a large number of microorganisms with a low rate of metabolic activity.

Both commercial and experimental controls showed dead microorganisms, but they were superimposed by a large amount of viable microorganisms. We believe that a considerable portion of these colonies, as demonstrated in several reports [49,50], were the compromised viable bacteria that were detected using live/dead staining, and they were not strong enough to grow on the agar plates. This was likely why the CFUs of the control groups were comparable to the Glass + NACP + 0% DMAHDM group, while the amount of green staining for the viable microorganisms (live and injured) under the microscope looked greater for control groups than for the Glass + NACP + 0% DMAHDM group. This outcome reveals the potential for developing a long-lasting and potent antibacterial cement to reduce the occurrence of secondary caries and increase the long-term service of fixed dental restorations [25,51].

Another potential technique to prevent secondary caries around dental restorations includes the addition of remineralizing agents to crown cement. The use of fluoride-releasing cements was suggested to neutralize the acidity caused by cariogenic species and remineralize the surrounding tooth structure. In addition, most of the fluoride-releasing materials in dentistry are associated with a burst release (a high amount of ion release) in the first 72 h followed by a low level of fluoride release. The low level of fluoride release may not ensure the required and long-lasting clinical outcomes [52,53]. The incorporation of NACP is a promising method for remineralizing and enhancing tooth structure [54]. The small particle size and high surface area are significant advantages of using NACP fillers. In the current analysis, a spray-drying technique was used to make NACP at a Ca^2+^/PO_4_^3−^ molar ratio of 1.5 and a particle size of 142 nm. In comparison to traditional CaP particles, NACP particles had a higher surface area of 17.76 m^2^/g, compared to about 0.5 m^2^/g [19]. This feature promotes the use of lower loads of the bioactive fillers to achieve substantial ion release.

In this investigation, to test the effect of DMAHDM on the Ca^2+^ and PO_4_^3−^ ion release, a range of 0%, 3%, and 5% DMAHDM was tested. All NACP and NACP + DMAHDM cement groups presented high levels of Ca^2+^ and PO_4_^3−^ ion release at a reasonably low level of NACP filler of 25%, which were considerably higher than the reported release from resin-based materials containing micro-sized CaP fillers [55]. Increasing the DMAHDM percentage from 3% to 5% did not significantly affect Ca^2+^ and PO_4_^3−^ ion release (*p* < 0.05). The lower level of the bioactive fillers created an opportunity for adding a higher level of reinforcing glass fillers to the resin matrix to enhance the mechanical properties.

For long-lasting fixed restoration, in addition to antibacterial and remineralization properties, excellent mechanical properties for the crown cement is an essential aspect. The present study showed that the NACP + DMAHDM crown resin cement achieved flexural strength comparable to the experimental control and higher than the commercial control cement. Conversely, the NACP + DMAHDM crown resin cement achieved elastic modulus results comparable to the experimental control and lower than the commercial control cement. The decreased elastic modulus could be due to the bioactive behavior of the base PEHB monomer of this new cement. The formulation’s chemical composition has a unique role for ion release, recharge, and re-release, which could give the final cement structure a more elastic outcome [32]. The decrease in the elastic modulus could be attributed to an insignificant effect in the dental crown performance due to the thin cement film thickness.

The novel NACP + DMAHDM crown resin cement showed film thickness within the recommended range of the ISO standard [56]. The good mechanical properties of the novel resin cement resulted from the addition of 20% glass particles as reinforcement fillers, which provided a thin film with an excellent fracture resistance.

Micro-mechanical interlocking is the key feature of the bonding of resin-based cement to dentin through resin penetration into the demineralized superficial layer of dentin [57]. Incomplete resin infiltration into dentinal tubules permits microleakage, reduce bond durability, and subsequently reduce the long-term success [58]. The bond strength of dental cement to dentin is a critical mechanical factor in the long-term success of the fixed restoration. Most of the stresses in the oral environment occur as a form of shear stress in the dental cement. Therefore, in this study, a dentin shear bond test was used to evaluate bond strength to dentin [59]. In the present study, all NACP + DMAHDM crown cement groups exhibited comparable results to the commercial control. There is a strong relationship between the severity of microleakage and the bonding strength of the cement. Excellent bonding properties contribute to increasing the longevity of the fixed restorations by reducing the microleakage and preventing secondary caries.

PMGDM and EBPADMA are the main monomers used in the novel cement. PMGDM is an acidic monomer that may have the ability to chelate with Ca^2+^ ions in the oral environment, and when exposed to an acidic environment, the ions could re-release when the bond between PMGDM and Ca^2+^ break down [32]. EBPADMA is a relatively hydrophobic monomer that has the ability to enhance the degree of conversion and flow of the cement [60]. HEMA was incorporated in the novel cement to improve hydrophilicity and significantly decrease the viscosity of the crown cement. BisGMA is a monomer with high molecular weight and has been shown to improve the cross-linking and bonding properties of the cement [61]. In the current analyses, increasing the BisGMA level from 4.85% to 6% resulted in a significant improvement in the dentin shear bond strength. However, to reduce any negative outcome on the cement durability, only a slight increase of BisGMA was used as it may increase the possible risk of resin-based material degradation and hydrolysis at the tooth-restoration interface [61].

As the commercial control cement is commonly used for fixed restoration cementation with high clinical success [62], the new NACP + DMAHDM crown cement with enhanced mechanical properties may also be suitable for similar use. Furthermore, the new cement has the additional advantages of long-lasting antibacterial activity throughout the contact inhibition of DMAHDM and the remineralization potential through the release of a high level of Ca^2+^ and PO_4_^3−^ ions. The novel NACP + DMAHDM crown cement was capable of being dual cured and they may find use in metaloceramic, ceramic, zirconia, and gold fixed restorations.

An antibacterial cement with a high level of Ca^2+^ and PO_4_^3−^ ion release can improve the long-term success of the crown cement by promoting remineralization and strengthening the tooth structures, as well as preventing demineralization and inhibiting secondary caries. Further studies are needed to examine the novel crown cement antibacterial effects on the killing of multispecies biofilms that are more clinically relevant. Furthermore, additional studies are needed to investigate the properties and performance of the new cement. This includes the degree of conversion, long-term mechanical performance, bond strength to commonly used crown materials, and long-term recharge and re-release of Ca^2+^ and PO_4_^3−^ ions.

## 5. Conclusions

This study developed a novel bioactive dental crown cement containing antibacterial monomer and calcium phosphate nanoparticles. Incorporating NACP and DMAHDM into the cement did not adversely affect the mechanical properties. The new NACP + DMAHDM crown cement has excellent potential for fixed restoration cementations, as it efficiently inhibited *S. mutans* biofilms commonly associated with secondary caries around dental restoration margins. Additionally, high levels of Ca^2+^ and PO_4_^3−^ ions released by the novel cement could promote remineralization and enhance tooth structures. The development of a new generation of resin-based crown cement with the combined effect of antibacterial and remineralizing properties could be the key to lengthen the survivability of fixed restorations. 

## Figures and Tables

**Table 1:** Chemical composition of RelyX luting cement.

Powder	Liquid
Fluoroaluminosilicate (FAS) glass Potassium persulfate Ascorbic acid Opacifying agent	Methacrylated polycarboxylic acid Water Hydroxyethyl methacrylate (HEMA) Tartaric acid

**Table 2:** Chemical composition of PEHB monomers with 0% dimethylaminohexadecyl methacrylate (DMAHDM).

**Part A Part B**			
**Chemical**	**Weight%**	**Chemical**	**Weight%**
Cumene hydroperoxide (CHP)	2%	Benzoyl thiourea (BTU)	1%
2,6-ditertbutyl-4-methylphenol (BHT)	0.05%	Camphorquinone (CQ)	0.4%
Pyromellitic glycerol dimethacrylate (PMGDM)	87.95%	Ethyl-4-*N*,*N*-dimethylaminobenzoate (4E)	1.6%
HEMA	10%	Ethoxylated bisphenol-A-dimethacrylate (EBPADMA)	77%
		Bisphenol A-glycidyl methacrylate (BisGMA)	10%
		HEMA	10%

**Table 3:** Chemical composition of PEHB monomers with 3% DMAHDM.

**Part A Part B**			
**Chemical**	**Weight%**	**Chemical**	**Weight%**
CHP	2%	BTU	1%
BHT	0.05%	CQ	0.4%
PMGDM	81.5%	4E	1.6%
HEMA	16.45%	EBPADMA	70.55%
		BisGMA	12%
		DMAHDM HEMA	10.9% 3.55%

**Table 4:** Chemical composition of PEHB monomers with 4% DMAHDM.

**Part A Part B**			
**Chemical**	**Weight%**	**Chemical**	**Weight%**
CHP	2%	BTU	1%
BHT	0.05%	CQ	0.4%
PMGDM	79.68%	4E	1.6%
HEMA	14.72%	EBPADMA	65.18%
EBPADMA	3.55%	BisGMA	12%
		DMAHDM HEMA	14.54% 5.28%

**Table 5:** Chemical composition of PEHB monomers with 5% DMAHDM.

**Part A Part B**			
**Chemical**	**Weight%**	**Chemical**	**Weight%**
CHP	2%	BTU	1%
BHT	0.05%	CQ	0.4%
PMGDM	77.95%	4E	1.6%
HEMA	16.45%	EBPADMA	63.45%
EBPADMA	3.55%	BisGMA	12%
		DMAHDM HEMA	18% 3.55%
